# Artificial intelligence in clinical decision support systems for oncology

**DOI:** 10.7150/ijms.77205

**Published:** 2023-01-01

**Authors:** Lu Wang, Xinyi Chen, Lu Zhang, Long Li, YongBiao Huang, Yinan Sun, Xianglin Yuan

**Affiliations:** 1 Department of Oncology, Tongji Hospital, Tongji Medical College, Huazhong University of Science and Technology, Wuhan, Hubei Province, China.; 2 Department of Cardiology, Tongji Hospital, Tongji Medical College, Huazhong University of Science and Technology, Wuhan, Hubei Province, China.

**Keywords:** AI, CDSS, WFO, tumor treatment, MDT

## Abstract

Artificial intelligence (AI) has been widely used in various medical fields, such as image diagnosis, pathological classification, selection of treatment schemes, and prognosis analysis. Especially in the image-aided diagnosis of tumors, the cooperation of human-computer interactions has become mature. However, the ethics of the application of AI as an emerging technology in clinical decision-making have not been fully supported, so the clinical decision support system (CDSS) based on AI technology has not fully realized human-computer interactions in clinical practice as the image-aided diagnosis system. The CDSS was currently used and promoted worldwide including Watson for Oncology, Chinese society of clinical oncology-artificial intelligence (CSCO AI) and so on. This paper summarized the applications and clarified the principle of AI in CDSS, analyzed the difficulties of AI in oncology decisions, and provided a reference scheme for the application of AI in oncology decisions in the future.

## Introduction

In the precision medicine era, the explosive growth of new drugs and approved indications has brought great challenges to the formulation of the best treatment scheme for different molecular phenotypes of different tumors 1-3. With the technical support of big data and machine learning, clinical decision support systems (CDSS) based on artificial intelligence (AI) came into being 4-6. By integrating different medical records, literature and clinical research data, the CDSS evaluates the drug efficacy, product accessibility, adverse reactions, the financial status of the patients and medical insurance types, and then provides individualized suggestions to help clinicians optimize treatment plans 7, 8. The applications of AI have expanded from solving daily life problems to medical professional fields, such as image diagnosis, pathological diagnosis, clinical treatment decision-making, prognosis analysis, and new drug screening 9, 10.

The Watson system of IBM has been applied to the fields of fashion, finance, medical treatment, tourism, law, education, transportation and so on. Especially in the field of cancer, Watson for Oncology (WFO, IBM Corporation, United States), as the earliest widely used CDSS 11, 12, has been gradually popularized all over the world in the fields of lung cancer, colon cancer, rectal cancer, breast cancer, gastric cancer and gynecological cancer 13. Based on the WFO system, the medical staff only need to input the structured data of the case. Within one minute, the system will output the most standard treatment method for the specific case and provide highly consistent evidence 11. The WFO has provided services to more than 70 municipal medical institutions and more than ten thousand patients 13. A CDSS called the Chinese society of clinical oncology-artificial intelligence (CSCO AI) system based on Chinese society of clinical oncology-breast cancer (CSCO BC) big data and CSCO BC guidelines had run into operation for three years nationwide 7. As of January 2022, excluding combined treatment regimens, there are currently 82 FDA drugs approved for breast cancer treatment 14. Breast cancer therapy is expected to accelerate the pace of treatment and shorten the cycle of treatment guideline changes. The Oncologists face a huge and rapidly expanding knowledge base challenge 15, 16. To mitigate this challenge, CSCO AI has now been applied to breast cancer treatment in the face of rapidly changing scientific evidence 17, 18, helping patients achieve personalized treatment based on drug approval and treatment guidelines. Other CDSSs, such as Archimedes IndiGO, Auminence, DiagnosisOne, and DXplain, have also gradually been developed and are beginning to be applied in the clinic 19, 20.

## Overview of the CDSS

The CDSS based on AI technology is essentially a computer program 8, 21. In the early stage, the knowledge base was constructed through the key and prognostic information in structured medical records or literatures. Then, a search engine was used to filter the best results from the knowledge base through key information and then feedback to users 22, machine learning algorithms include logistic regression, support vector machine, artificial neural network, deep learning and so on. Due to the particularity of clinical application and the development of CDSS technology, unsupervised models are mainly used to operate. AI technology allows computers to simulate human reasoning thinking, collect and express the learned knowledge content by generating suggestions, reducing the dependence on memory, the decision error rate and the response time. It also helps make reasonable and safe decisions in clinical practice to ensure clinical safety, quality and improve the treatment efficiency 7, 11.

## Composition and treatment judgment of the CDSS

As one of the earliest CDSS in the world, WFO has indexed and stored test cases, documents, protocols and medical records from experts at the Memorial Sloan Kettering Cancer Center (MSKCC) and applied them to specific cases after being processed by a computational reasoning method 23, 24. In addition, unlike the CSCO AI, WFO updates the latest diagnosis and treatment information every one to two months and for understudied medical records, WFO also describes unsupported case information. When enter case information that WFO does not support, it would not process the case and return any suggestions 25. WFO's treatment recommendations are divided into three groups and have corresponding labels: green represents “recommended treatment”, which has a strong evidence base, orange represents “consideration treatment”, and according to their clinical judgment, doctors can consider it as a suitable substitute, while red represents non-recommended treatment, which means due to specific contraindications or strong evidence against their use. If WFO is not aware of it at the time of the analysis, the treatment recommended by the oncology committee is classified as “unavailable” 26.

**Figure 1:**
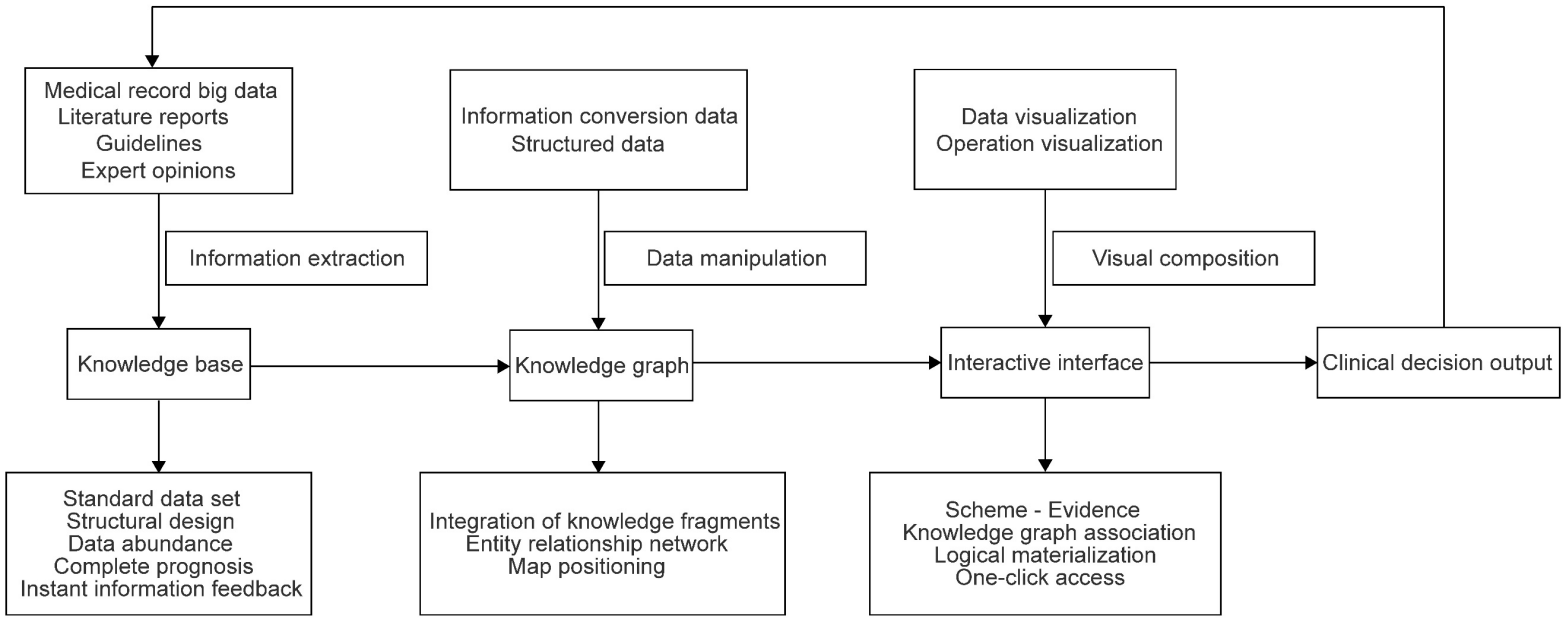
** Flow chart of the data operation in the CDSS.** The data in the flow chart of the CDSS were operated according to the knowledge base, knowledge graph, interactive interface and clinical decision output sites.

Different from WFO, CSCO AI system was established under the CSCO platform by using the CSCO database, guidelines. The CSCO AI system mainly built different knowledge maps based on the schemes in the CSCO guidelines. When doctors search for relevant information, it locates the knowledge map and output the results according to the key information. Similarly, it is also updated in real time with the guidelines to ensure the timeliness of the system, as shown in Figure 1. In addition to incorporating guidelines, medical records and literature data, CSCO AI also integrates medical insurance, clinical research and other data to make it more in line with the actual situation in China and to ensure that users can obtain more information with one click 7. For a self-assessment of the decision-making scheme, CSCO AI adopts a quantitative scoring method, if the decision-making scheme conforms to the national comprehensive cancer network (NCCN) guideline class 1 evidence recommended by the high-level guidelines or the CSCO BC guideline class I recommendation and conforms to clinical practice, it is fully compliant, and 3 points can be given; if it meets the high-level recommendation but does not meet the clinical practice, or meets the class 2A evidence of NCCN guidelines or the class II recommendation of CSCO BC guidelines and meets the clinical practice, it is in high-level compliance, and 2 points can be given. If it does not conform to the guidelines but conforms to clinical practice or only conforms to class 2B evidence of the NCCN guidelines or class III recommendations of the CSCO BC guidelines, 1 point can be given. If it does not comply with the guidelines and does not comply with clinical practice, 0 points will be given 7.

## Application characteristics of the CDSS

**Figure 2:**
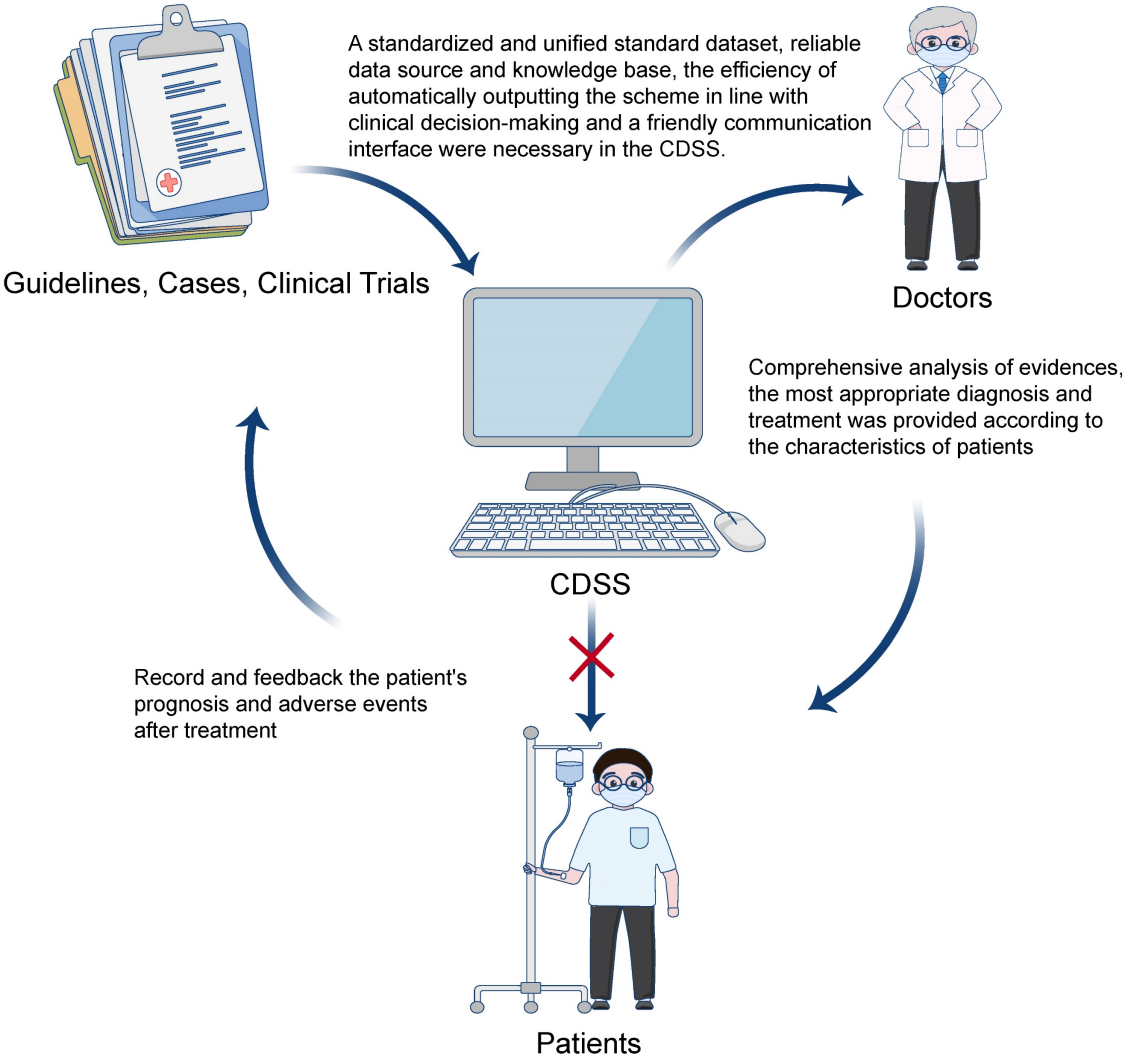
** The applications of CDSS in clinical work.** The knowledge base was constructed through the key and prognostic information in structured medical records, literature and guidelines. The search engine was used to filter the best results from the knowledge base through key information and feedback to the doctors. The doctor could make a clinical decision based on the CDSS suggestion and their clinical experience to treat patients, and the prognostic information of patients should be studied by the knowledge base that forms a positive cycle.

Based on the working principle, CDSS has several characteristics. First, the development knowledge base of the CDSS mainly comes from the big data of clinical guidelines or case composition included in high-level clinical research 27, 28. Second, for the CDSS developed based on a guideline, convenience is improved at the cost of reducing its reliability 7, 29-31. Third, the updating speed and the abundance of information sources of the knowledge base can affect the heterogeneity of conclusions between the CDSS and multi-disciplinary team (MDT) 32. Fourth, the CDSS can guide inexperienced doctors and interns 33. However, the CDSS can only be regarded as a member of the MDT team as shown in Figure 2. After more than 20 years of development, the CDSS has played an important role in disease management, radiation dose calculation, image analysis, blood bank systems, nursing and other fields 34-36. At present, most clinical studies on the reliability of CDSS has compared the schemes of MDT and MDT plus CDSS to explore the help provided by the AI system to doctors at different levels and for different diseases, which is closely related to the ethics of AI technology in clinical diagnosis and treatment decision-making 37.

For the CSCO AI system, the establishment of a standardized and unified standard dataset, reliable data source and knowledge base, the efficiency of automatically outputting the scheme in line with clinical decision-making and a friendly communication interface are important directions to improve the reliability of the CDSS 7. The knowledge base is the cornerstone of the CSCO AI system. System learning, testing and verification should be based on the knowledge base. The richness and representativeness of the knowledge base content directly affect the reliability of the system. The knowledge base of the CSCO AI mainly comes from clinical data and guideline evidence. The former is mostly semi-structured or unstructured, and the latter is structured data 38. For the individual characteristics of patients in the literature (such as gender, age, PET, CT, MRI, blood tests, body fluid tests, stool tests, different tumor node metastasis (TNM) stages, molecular typing, past history, adverse reactions, etc.), mapping abstract data into graphic elements, supplemented by human-computer interactions, play the very important role in the application of knowledge retrieval, question and answer, knowledge recommendation, knowledge visualization and so on 39, 40. It also provides an ideal technical method to solve the problem of "data islands", which is helpful to realize the integration of knowledge resources and improve the ability of knowledge services. For text-based data, it is necessary to conduct text analysis under the specifications of medical standard terms, such as semantic annotation and association analysis, to obtain all conceptual datasets about tumor diagnosis and treatment 7.

AI in the medical field usually focuses on acquiring knowledge from unstructured data, such as text (using natural language processing) or large structured datasets (using machine learning methods) 41-43. After storing, indexing and delineating this knowledge, it uses computational reasoning methods to apply it to specific situations, generate and evaluate hypotheses, and then provide an evaluation for doctors 44, 45. There are several diagnostic criteria for cancer, such as CancerLinq 46 and a system called OncoDoc 47. Through “reading” the literature, protocols and medical records, and learning from MSKCC test cases and experts, WFO obtains a large amount of knowledge as a preliminary knowledge base 23, 24. At the same time, WFO also provides evidence to support recommended treatments, as well as specific case clinical trials, prescription information, potential adverse reactions related to treatment 11. At present, WFO can provide rapid and accurate treatment suggestions for most cancer patients, compared the treatment regimens recommended for breast cancer cases between the Manipal multidisciplinary tumor board (MMDT) and WFO, the overall treatment concordance is up to 93% 11. It can also play an important role in reducing doctors' workload and training young doctors. In addition, WFO would regulate the treatment of cancers nationwide and enhance the trust between doctors and patients, especially in rural hospitals 13, 48.

Moreover, a CDSS should provide detailed evidence to support its recommendations, which is based on the credibility of the relevant literature 11. Doctors can review the relevant evidence and judge whether it is applicable to the current case. When they choose to treat according to the recommended scheme, they also provide other information, such as the survival rate, incidence of adverse reactions, evidence supporting the recommended treatment, possible specific cases and clinical trials, prescription information, potential adverse reactions related to treatment to help doctors evaluate the efficacy and risk of the whole scheme. The CDSS can significantly shorten the time for junior doctors to consult the relevant literature and improve their ability to make accurate diagnosis and treatment suggestions in a short time. At the same time, it could eliminate the time cost by patients visiting top hospitals and help patients obtain the best treatment as soon as possible. Finally, the CDSS may solve the problem of doctor-patient trust. In contemporary China, patients' distrust of doctors is increasing due to many reasons, such as a shortage of funds, excessive market-oriented operation, limitations of medical insurance reimbursement and a large number of nonneutral reports of health events in the media 49-51. In addition, patients often suspect doctors of engaging in overtreatment 52. The CDSS has no personal preferences. Therefore, it can be regarded as an objective and fair decision-making system and win the trust of patients. Cancer patients do not have to visit multiple experts to find a treatment they think is fair 53, 54.

## The difficulties of application and development of the CDSS

With the increasing popularity of WFO all over the world, in practice, many local doctors and medical institutions question the extent to whether WFO is suitable for cancer patients in their country. The problem is mainly emphasized in two aspects. On the one hand, a percentage of cancer cases is not supported by WFO. If the proportion of unsupported cases is very large (e.g., more than 50%), the total application value of WFO will be affected. On the other hand, in the supported cases, are the recommendations of WFO consistent with those of MDT? In addition, how can we make the decision-making opinions put forward by WFO be more suitable for the local people 37?

In a study on lung cancer, among the medical records supported by the study on patients with isolated metastasis and patients with mutations in the driving gene of cancer progression, WFO did not support 18.1% of cases, among which 42% progressed after targeted therapy. The reason may be that the EGFR gene mutation phenotype of lung cancer in China is very different from that in the western countries. The mutation rate of the EGFR gene in patients with lung cancer is approximately 15% in Europe and the United States, while the probability of this mutation in China is 50% or higher 55-57. Additionally, the drugs circulating in the market vary from country to country, such as immune checkpoint inhibitors involving PD-1 and PD-L1 antibodies. Patients' preferences, finance and medical insurance also need to be taken into account, which would eventually affect the consistency. Inconsistencies between WFO and MDT occurred in 7% of cases. 23% of these cases was due to differences in regulatory approval processes between countries, which can be remedied by incorporating locally approved therapies into the knowledge base of the expert system. The inconsistency may also be due to differences in treatment methods in patient subgroups affected by demographic characteristics such as the comorbidity burden, patient preference and social support system 58-61.

There are several difficulties in the current clinical research on CDSS. The sample size of the existing research is very small, and most studies do not mention unsupported cases, which would affect the efficiency of clinical use 62, 63. Although the doctors who input cases into the CDSS are familiar with the system and the key elements to be extracted during the chart review process, the quality and repeatability of these tasks have not been formally tested, which may affect the recommendations of the CDSS. Since the CDSS provides evidence for its decision-making, oncologists could examine the evidence and consider the basis of the CDSS recommendations. An unexpected recommendation by the CDSS may prompt clinicians to examine their evidence and reconsider it according to the evaluation of the MDT. Although the use of the CDSS may also help to ensure that treatment is more standardized at the appropriate time, when personal preferences or well-known cognitive biases dominate personal decision-making, there may be treatment options that are not objectively standardized 64, 65. Finally, lack of consistency does not mean the recommendations of MDT or CDSS are “uncorrected”. There are many effective explanations for differences, such as the differences in treatment methods between comorbid or aging patients 66-68.

## Development direction of the CDSS

There is still much room for the development and improvement of the CDSS in the future. First, the update rate of the knowledge base needs to be further improved. Second, the abundance of input information and structured knowledge base information during training will reduce unsupported cases. Third, the CDSS feedback port should be more open, when there is no support case according to the treatment decision put forward by the CDSS, it should be discussed by the MDT and fed back to the CDSS for learning. After the treatment plan is proposed according to the decision, the dynamic changes in treatment also need to be fed back to the CDSS for learning. Fourth, the recommendation level of the CDSS for treatment decision-making should be quantified and evaluated according to the abundance of evidence. Fifth, when different countries introduce the CDSS, diagnosis and treatment equipment and drugs should be localized to improve the accessibility of the CDSS.

The storage capacity of an AI-based CDSS is much stronger than that of the human brain. It can quickly collect and sort stored information to draw accurate conclusions faster than humans, such as diagnostic radiology and pathological imaging systems 69-71. However, to adapt to a real native medical environment, the CDSS must be significantly improved. After obtaining ethical approval, the medical data of patients should be standardized and shared nationwide, and the follow-up system should be improved to obtain the complete information of patients. In different countries, the unique medical data warehouse should be established for the CDSS research. These data should be combined with international guidelines and medical systems in different countries to enable the CDSS to give full potential to serve patients in different countries 11, 72, 73.

It should be emphasized that the CDSS cannot replace oncologists at present 74, 75. It should be positioned as a good assistant or teacher for young doctors 13. And there is another angle that is Collaborative intelligence, human and AI working synergistically. With the rapid development of CDSS, the CDSS for cervical cancer prevention automatically provides recommendations, accuracy of which is improved to 93% vs. the expert clinician as per the American Society of Colposcopy and Cervical Pathology (ASCCP) guidelines 76. some doctors blindly follow the suggestions of the CDSS and think it will replace them in the future. However, just 60% of treatment pairs for breast, lung, colon, and rectal cancers are identical or equally acceptable, with 70% of WFO therapeutic options identical to, or acceptable alternatives to, Bumrungrad International Hospital therapy. What's more, colorectal cancers exhibited the highest proportion of identical or equally acceptable treatments; however, stage IV cancers demonstrate the lowest. Therefore, collaborative intelligence already exists and very likely to dominate in the future, the CDSS that could support, rather than replace the clinicians provides therapeutic options which are generally consistent with recommendations 74. Also, medicine is not only a science but it also involves other aspects, such as social and psychological factors 77, 78. Doctors must consider individualized measures for different patients, even those with the same diagnosis. When using the CDSS, oncologists are required to confirm whether patients can tolerate surgery or radiotherapy, or whether there is any emergency. The treatment plan could only continue if this information is confirmed. After it provides treatment suggestions, the most appropriate treatment scheme according to the patient's physical and mental status, financial status, complications and willingness to receive treatment.

In conclusion, the coincidence rate between the treatment decision made by the CDSS and the treatment decision of the MDT would be higher as AI technology continues to mature although the availability of drug treatment, professional treatment guidelines and the judgment of expert training will affect consistency. The AI-based CDSS may have a wide range of value in providing tumor treatment suggestions, especially at the hospitals where expert resources are not easy to obtain 11. Before the CDSS acts as a doctor's assistant, scientists should continue to explore and innovate to further promote medical progress 14.

## Author Contributions

**Yinan Sun and Lu Wang**: Writing - original draft. **Lu Wang and Xinyi Chen**: Writing - review & editing. **Yongbiao Huang, Lu Zhang, Long Li and Xianglin Yuan**: Writing - review & editing, Supervision.

## Abbreviations

AI: Artificial intelligence
CDSS: clinical decision support system
CSCO AI: Chinese society of clinical oncology-artificial intelligence
WFO: Watson for Oncology
CSCO BC: Chinese society of clinical oncology-breast cancer
MSKCC: Memorial Sloan Kettering Cancer Center
NCCN: national comprehensive cancer network
MDT: multi-disciplinary team
TNM: tumor node metastasis
MMDT: Manipal multidisciplinary tumor board
ASCCP: American Society of Colposcopy and Cervical Pathology (ASCCP)

## References

[1] Park K (2019). A review of computational drug repurposing. Transl Clin Pharmacol.

[2] Berger MF, Mardis ER (2018). The emerging clinical relevance of genomics in cancer medicine. Nat Rev Clin Oncol.

[3] Jonna S, Subramaniam DS (2019). Molecular diagnostics and targeted therapies in non-small cell lung cancer (NSCLC): an update. Discov Med.

[4] Cricelli I, Marconi E, Lapi F (2022). Clinical Decision Support System (CDSS) in primary care: from pragmatic use to the best approach to assess their benefit/risk profile in clinical practice. Curr Med Res Opin.

[5] Veggiotti N, Sacchi L, Peleg M (2021). Enhancing the IDEAS Framework with Ontology: Designing Digital Interventions for Improving Cancer Patients' Wellbeing. AMIA Annu Symp Proc.

[6] Hadjiiski LM, Cha KH, Cohan RH, Chan HP, Caoili EM, Davenport MS (2020). Intraobserver Variability in Bladder Cancer Treatment Response Assessment With and Without Computerized Decision Support. Tomography.

[7] Li JB, Jiang ZF (2020). [Establishment and its application of Chinese society of clinical oncology artificial intelligence system (CSCO AI)]. Zhonghua Yi Xue Za Zhi.

[8] Shortliffe EH, Sepulveda MJ (2018). Clinical Decision Support in the Era of Artificial Intelligence. JAMA.

[9] Kawamoto A, Takenaka K, Okamoto R, Watanabe M, Ohtsuka K (2022). A Systematic Review of Artificial Intelligence-Based Image Diagnosis for Inflammatory Bowel Disease. Dig Endosc.

[10] Cao R, Gong L, Dong D (2021). Pathological diagnosis and prognosis of Gastric cancer through a multi-instance learning method. EBioMedicine.

[11] Somashekhar SP, Sepulveda MJ, Puglielli S, Norden AD, Shortliffe EH, Rohit Kumar C (2018). Watson for Oncology and breast cancer treatment recommendations: agreement with an expert multidisciplinary tumor board. Ann Oncol.

[12] Trivedi H, Mesterhazy J, Laguna B, Vu T, Sohn JH (2018). Automatic Determination of the Need for Intravenous Contrast in Musculoskeletal MRI Examinations Using IBM Watson's Natural Language Processing Algorithm. J Digit Imaging.

[13] Liu C, Liu X, Wu F, Xie M, Feng Y, Hu C (2018). Using Artificial Intelligence (Watson for Oncology) for Treatment Recommendations Amongst Chinese Patients with Lung Cancer: Feasibility Study. J Med Internet Res.

[14] Institute NC (2022). Drugs Approved for Breast Cancer. National Institutes of Health.

[15] Beyer T, Bidaut L, Dickson J, Kachelriess M, Kiessling F, Leitgeb R (2020). What scans we will read: imaging instrumentation trends in clinical oncology. Cancer Imaging.

[16] Bright TJ, Wong A, Dhurjati R, Bristow E, Bastian L, Coeytaux RR (2012). Effect of clinical decision-support systems: a systematic review. Ann Intern Med.

[17] Sharing Clinical Trial Data (2015). Maximizing Benefits, Minimizing Risk. Washington (DC).

[18] Taichman DB, Backus J, Baethge C, Bauchner H, de Leeuw PW, Drazen JM (2016). Sharing Clinical Trial Data-A Proposal from the International Committee of Medical Journal Editors. N Engl J Med.

[19] Muller L, Gangadharaiah R, Klein SC, Perry J, Bernstein G, Nurkse D (2019). An open access medical knowledge base for community driven diagnostic decision support system development. BMC Med Inform Decis Mak.

[20] Elkin PL, Liebow M, Bauer BA, Chaliki S, Wahner-Roedler D, Bundrick J (2010). The introduction of a diagnostic decision support system (DXplain) into the workflow of a teaching hospital service can decrease the cost of service for diagnostically challenging Diagnostic Related Groups (DRGs). Int J Med Inform.

[21] Yun HJ, Kim HJ, Kim SY, Lee YS, Lim CY, Chang HS (2021). Adequacy and Effectiveness of Watson For Oncology in the Treatment of Thyroid Carcinoma. Front Endocrinol (Lausanne).

[22] Miller DD, Brown EW (2018). Artificial Intelligence in Medical Practice: The Question to the Answer?. Am J Med.

[23] Choi YI, Chung JW, Kim KO, Kwon KA, Kim YJ, Park DK (2019). Concordance Rate between Clinicians and Watson for Oncology among Patients with Advanced Gastric Cancer: Early, Real-World Experience in Korea. Can J Gastroenterol Hepatol.

[24] Lee WS, Ahn SM, Chung JW, Kim KO, Kwon KA, Kim Y (2018). Assessing Concordance With Watson for Oncology, a Cognitive Computing Decision Support System for Colon Cancer Treatment in Korea. JCO Clin Cancer Inform.

[25] Jie Z, Zhiying Z, Li L (2021). A meta-analysis of Watson for Oncology in clinical application. Sci Rep.

[26] Zhang W, Qi S, Zhuo J, Wen S, Fang C (2020). Concordance Study in Hepatectomy Recommendations Between Watson for Oncology and Clinical Practice for Patients with Hepatocellular Carcinoma in China. World J Surg.

[27] Kindle RD, Badawi O, Celi LA, Sturland S (2019). Intensive Care Unit Telemedicine in the Era of Big Data, Artificial Intelligence, and Computer Clinical Decision Support Systems. Crit Care Clin.

[28] Chien SC, Chen YL, Chien CH, Chin YP, Yoon CH, Chen CY (2022). Alerts in Clinical Decision Support Systems (CDSS): A Bibliometric Review and Content Analysis. Healthcare (Basel).

[29] Kilsdonk E, Peute LW, Jaspers MW (2017). Factors influencing implementation success of guideline-based clinical decision support systems: A systematic review and gaps analysis. Int J Med Inform.

[30] Zollner JP, Wolking S, Weber Y, Rosenow F (2021). [Decision support systems, assistance systems and telemedicine in epileptology]. Nervenarzt.

[31] Zhao W, Jiang X, Wang K, Sun X, Hu G, Xie G (2020). Construction of Guideline-Based Decision Tree for Medication Recommendation. Stud Health Technol Inform.

[32] Pluyter JR, Jacobs I, Langereis S, Cobben D, Williams S, Curfs J (2020). Looking through the eyes of the multidisciplinary team: the design and clinical evaluation of a decision support system for lung cancer care. Transl Lung Cancer Res.

[33] Papandria D, Fisher JG, Kenney BD, Dykes M, Nelson A, Diefenbach KA (2020). Orientation in Perpetuity: An Online Clinical Decision Support System for Surgical Residents. J Surg Res.

[34] Mebrahtu TF, Skyrme S, Randell R, Keenan AM, Bloor K, Yang H (2021). Effects of computerised clinical decision support systems (CDSS) on nursing and allied health professional performance and patient outcomes: a systematic review of experimental and observational studies. BMJ Open.

[35] Jung H, Park HA (2019). Development and Evaluation of a Prototype CDSS for Fall Prevention. Stud Health Technol Inform.

[36] Campion TR Jr, May AK, Waitman LR, Ozdas A, Lorenzi NM, Gadd CS (2011). Characteristics and effects of nurse dosing over-rides on computer-based intensive insulin therapy protocol performance. J Am Med Inform Assoc.

[37] Zhou N, Zhang CT, Lv HY, Hao CX, Li TJ, Zhu JJ (2019). Concordance Study Between IBM Watson for Oncology and Clinical Practice for Patients with Cancer in China. Oncologist.

[38] Kristianson KJ, Ljunggren H, Gustafsson LL (2009). Data extraction from a semi-structured electronic medical record system for outpatients: a model to facilitate the access and use of data for quality control and research. Health Informatics J.

[39] Barua A, Watson K, Plesons M, Chandra-Mouli V, Sharma K (2020). Adolescent health programming in India: a rapid review. Reprod Health.

[40] Gionfriddo MR, Duboski V, Middernacht A, Kern MS, Graham J, Wright EA (2021). A mixed methods evaluation of medication reconciliation in the primary care setting. PLoS One.

[41] Jiang F, Jiang Y, Zhi H, Dong Y, Li H, Ma S (2017). Artificial intelligence in healthcare: past, present and future. Stroke Vasc Neurol.

[42] Moldwin A, Demner-Fushman D, Goodwin TR (2021). Empirical Findings on the Role of Structured Data, Unstructured Data, and their Combination for Automatic Clinical Phenotyping. AMIA Jt Summits Transl Sci Proc.

[43] Bettencourt-Silva J, Mulligan N, Cullen C, Kotoulas S (2018). Bridging Clinical and Social Determinants of Health Using Unstructured Data. Stud Health Technol Inform.

[44] Dong C, Wang Y, Zhou J, Zhang Q, Wang N (2020). Differential Diagnostic Reasoning Method for Benign Paroxysmal Positional Vertigo Based on Dynamic Uncertain Causality Graph. Comput Math Methods Med.

[45] Tsafnat G, Coiera EW (2009). Computational reasoning across multiple models. J Am Med Inform Assoc.

[46] Schorer AE, Moldwin R, Koskimaki J, Bernstam EV, Venepalli NK, Miller RS (2022). Chasm Between Cancer Quality Measures and Electronic Health Record Data Quality. JCO Clin Cancer Inform.

[47] Redjdal A, Bouaud J, Guezennec G, Gligorov J, Seroussi B (2021). Reusing Decisions Made with One Decision Support System to Assess a Second Decision Support System: Introducing the Notion of Complex Cases. Stud Health Technol Inform.

[48] Zhao X, Zhang Y, Ma X, Chen Y, Xi J, Yin X (2020). Concordance between treatment recommendations provided by IBM Watson for Oncology and a multidisciplinary tumor board for breast cancer in China. Jpn J Clin Oncol.

[49] Zhou P, Grady SC (2016). Three modes of power operation: Understanding doctor-patient conflicts in China's hospital therapeutic landscapes. Health Place.

[50] Chan CS (2018). Mistrust of physicians in China: society, institution, and interaction as root causes. Dev World Bioeth.

[51] Wehkamp KH, Naegler H (2017). The Commercialization of Patient-Related Decision Making in Hospitals. Dtsch Arztebl Int.

[52] Sugitani I, Ito Y, Takeuchi D, Nakayama H, Masaki C, Shindo H (2021). Indications and Strategy for Active Surveillance of Adult Low-Risk Papillary Thyroid Microcarcinoma: Consensus Statements from the Japan Association of Endocrine Surgery Task Force on Management for Papillary Thyroid Microcarcinoma. Thyroid.

[53] Rajput VK, Dowie J, Kaltoft MK (2020). Are Clinical Decision Support Systems Compatible with Patient-Centred Care?. Stud Health Technol Inform.

[54] Van Dort BA, Zheng WY, Baysari MT (2019). Prescriber perceptions of medication-related computerized decision support systems in hospitals: A synthesis of qualitative research. Int J Med Inform.

[55] Li T, Kung HJ, Mack PC, Gandara DR (2013). Genotyping and genomic profiling of non-small-cell lung cancer: implications for current and future therapies. J Clin Oncol.

[56] Zhou C (2014). Lung cancer molecular epidemiology in China: recent trends. Transl Lung Cancer Res.

[57] Yao S, Wang R, Qian K, Zhang Y (2020). Real world study for the concordance between IBM Watson for Oncology and clinical practice in advanced non-small cell lung cancer patients at a lung cancer center in China. Thorac Cancer.

[58] Zaman SB, Evans RG, Singh R, Singh A, Singh P, Singh R (2021). Feasibility of community health workers using a clinical decision support system to screen and monitor non-communicable diseases in resource-poor settings: study protocol. Mhealth.

[59] Linkens A, Milosevic V, van Nie N, Zwietering A, de Leeuw PW, van den Akker M (2022). Control in the Hospital by Extensive Clinical rules for Unplanned hospitalizations in older Patients (CHECkUP); study design of a multicentre randomized study. BMC Geriatr.

[60] Schouten BC, Hoogstraten J, Eijkman MA (2003). Patient participation during dental consultations: the influence of patients' characteristics and dentists' behavior. Community Dent Oral Epidemiol.

[61] Weltermann B, Kersting C (2016). Feasibility study of a clinical decision support system for the management of multimorbid seniors in primary care: study protocol. Pilot Feasibility Stud.

[62] Rahimi R, Kazemi A, Moghaddasi H, Arjmandi Rafsanjani K, Bahoush G (2018). Specifications of Computerized Provider Order Entry and Clinical Decision Support Systems for Cancer Patients Undergoing Chemotherapy: A Systematic Review. Chemotherapy.

[63] van Wijk Y, Halilaj I, van Limbergen E, Walsh S, Lutgens L, Lambin P (2019). Decision Support Systems in Prostate Cancer Treatment: An Overview. Biomed Res Int.

[64] van Baalen S, Boon M, Verhoef P (2021). From clinical decision support to clinical reasoning support systems. J Eval Clin Pract.

[65] Kuusisto F, Dutra I, Elezaby M, Mendonca EA, Shavlik J, Burnside ES (2015). Leveraging Expert Knowledge to Improve Machine-Learned Decision Support Systems. AMIA Jt Summits Transl Sci Proc.

[66] Beeler PE, Bates DW, Hug BL (2014). Clinical decision support systems. Swiss Med Wkly.

[67] Ankolekar A, van der Heijden B, Dekker A, Roumen C, De Ruysscher D, Reymen B (2022). Clinician perspectives on clinical decision support systems in lung cancer: Implications for shared decision-making. Health Expect.

[68] Bowles KH, Ratcliffe SJ, Holmes JH, Keim S, Potashnik S, Flores E (2019). Using a Decision Support Algorithm for Referrals to Post-Acute Care. J Am Med Dir Assoc.

[69] Gore JC (2020). Artificial intelligence in medical imaging. Magn Reson Imaging.

[70] Niazi MKK, Parwani AV, Gurcan MN (2019). Digital pathology and artificial intelligence. Lancet Oncol.

[71] Camacho J, Zanoletti-Mannello M, Landis-Lewis Z, Kane-Gill SL, Boyce RD (2020). A Conceptual Framework to Study the Implementation of Clinical Decision Support Systems (BEAR): Literature Review and Concept Mapping. J Med Internet Res.

[72] Kux BR, Majeed RW, Ahlbrandt J, Rohrig R (2017). Factors Influencing the Implementation and Distribution of Clinical Decision Support Systems (CDSS). Stud Health Technol Inform.

[73] Ali T, Hussain M, Ali Khan W, Afzal M, Lee S (2013). Authoring tool: acquiring sharable knowledge for Smart CDSS. Annu Int Conf IEEE Eng Med Biol Soc.

[74] Suwanvecho S, Suwanrusme H, Jirakulaporn T, Issarachai S, Taechakraichana N, Lungchukiet P (2021). Comparison of an oncology clinical decision-support system's recommendations with actual treatment decisions. J Am Med Inform Assoc.

[75] Xu F, Sepulveda MJ, Jiang Z, Wang H, Li J, Liu Z (2020). Effect of an Artificial Intelligence Clinical Decision Support System on Treatment Decisions for Complex Breast Cancer. JCO Clin Cancer Inform.

[76] Ravikumar KE, MacLaughlin KL, Scheitel MR, Kessler M, Wagholikar KB, Liu H (2018). Improving the Accuracy of a Clinical Decision Support System for Cervical Cancer Screening and Surveillance. Appl Clin Inform.

[77] Klein WM, Shepperd JA, Suls J, Rothman AJ, Croyle RT (2015). Realizing the promise of social psychology in improving public health. Pers Soc Psychol Rev.

[78] Wahl HW, Diegelmann M (2015). [Perspectives of psychological aging research]. Urologe A.

